# Wide Dynamic Range, Angle-Sensing, Long-Wave Infrared Detector Using Nano-Antenna Arrays

**DOI:** 10.1038/s41598-020-59440-2

**Published:** 2020-02-12

**Authors:** Elham Mohammadi, Mohammad Ghaffari, Nader Behdad

**Affiliations:** University of Wisconsin-Madison, Department of Electrical and Computer Engineering, Madison, WI 53706 USA

**Keywords:** Electrical and electronic engineering, Imaging and sensing

## Abstract

We present a new technique for designing angle-sensing, long-wave infrared (LWIR) detectors. Angle detection in the proposed detector is achieved by measuring the ratio of the absorbed power in two closely-spaced, directive infrared antennas. Each directive LWIR antenna is in the form of a three-element Yagi-Uda array sharing a common reflector element with its neighbor. The structure of each antenna is optimized to act both as the collector of the infrared energy from the desired direction and as a distributed bolometer that senses the received radiation. The resistivity of each bolometer-antenna changes as a function of the absorbed power by the antenna. This change of resistance is sensed by biasing each antenna with a constant DC voltage and measuring the change of current passing through the antenna. Following this approach, by measuring the ratio of the resistance change in the two antennas, the angle of arrival of the LWIR signal can be determined. We present the design, fabrication, and measurement results of an angle-sensing detector optimized to operate at the wavelength of *λ* = 10.6 *μ*m. The proposed detector has subwavelength dimensions occupying an aperture having dimensions of approximately 0.6 *λ*_0_ × 0.4*λ*_0_. The response of the detector was measured and shows the angle sensing dynamic range of 22 dB within the field of view of ±60°.

## Introduction

Infrared (IR) detectors, working at infrared wavelengths between 8–12 *μ*m, are in high demand since they can detect the thermal energy radiated from the objects at room temperature^1^. Commercially-available IR detectors are divided into two broad groups of photon detectors and thermal detectors. Photon detectors work based on the photon absorption in semiconductors, while in thermal detectors the absorbed incident radiation increases the temperature of the device, which results in variations of physical properties that can be sensed through external methods^2^. Photon detectors have better detectivity and faster response times. However, thermal detectors can work at room temperature but they usually suffer from lower response times and detectivities^2^. Infrared antennas can be coupled to various types of infrared detectors such as microbolometers^3–5^, thermocouples^6,7^, metal-insulator-metal (MIM) diodes^8,9^, and Schottky diodes^10^ to achieve faster responses compared to those of conventional thermal IR detectors^11,12^. In these devices, the sensitivity of the antennas to the incoming electromagnetic (EM) radiation results in an induced current, which is proportional to the intensity of the EM wave in the antenna. This current is detected by the IR sensor coupled to the antenna. If the sensor is thermal in nature (e.g., a bolometer), the current dissipated in it raises its temperature resulting in a change of resistance that can be sensed by appropriately biasing the detector. Other detectors such as MIM diodes can also be coupled to such antennas. In such a situation the detection of the IR radiation can be performed through the nonlinear process of rectification. In addition to having faster response times, which has been achieved by employing smaller detectors, antenna-coupled infrared detectors are sensitive to other attributes of the incoming EM wave, such as polarization, spectral content, and directional angular patterns^13,14^. Taking advantage of these other attributes of the incoming wave in imaging applications and target tracking systems offers additional information that can be used to increase the performances of these systems, especially in highly degraded environments^15^.

The sensitivity of an antenna to various characteristics of the incoming wave such as the angle of incidence and polarization is determined by the topology of the antenna and the electrical dimensions (physical dimensions normalized to wavelength) of its aperture. For instance, dipole^16^, bowtie^14^, and slot antennas^17^ are sensitive to linearly-polarized (i.e., vertical or horizontal polarization) waves while spiral antennas are most sensitive to circularly-polarized waves (i.e., right-hand or left-hand circular polarization)^14^. Therefore, one or more of these antennas could be used to detect the state of polarization of the incoming wave. Furthermore, the sensitivity of an antenna to the direction of arrival of an electromagnetic wave is primarily determined by the shape of its radiation pattern. Using antenna arrays is a good way to synthesize the desired radiation pattern shape^18^. For example, Middlebrook *et al*. used fixed-beam antenna arrays to achieve directional radiation patterns at IR wavelengths^19^. In their work, they used various configurations of two-element dipole antenna arrays coupled to bolometer as an IR detector. In order to achieve the desired directional radiation pattern, they changed the position of bolometer with respect to the two dipole antennas^20^. To use such arrays in angle sensing applications, one array should be mechanically rotated to scan various directions or multiple such arrays with various radiation patterns should be used. However, the relatively large spacing between the elements of each array (approximately one wavelength^20^) in conjunction with the requirements of using multiple arrays significantly increases the overall dimensions of angle-sensing detectors that use this concept.

Recently, we used the concept of biomimetic antenna arrays^21–23^ to design an angle-sensing infrared detector with sub-wavelength dimensions. This detector uses two strongly-coupled dipole antennas whose natural mutual coupling is augmented by using an external coupling network. This coupled-antenna IR detector was designed to serve two important functions. First, the detector collects the maximum amount of power from an incoming IR wave. Secondly, the coupled system was designed to make the output power ratio of the two antennas a one-to-one function of the angle of incidence of the incoming IR radiation. This way, by simply performing two amplitude measurements, the angle of arrival of the IR radiation may be resolved. Fabricated prototypes of this angle-sensing detector were shown to have a dynamic range of 10 dB while occupying an aperture size of 0.55*λ* × 0.7*λ*^21^. The dynamic range of this detector is primarily determined by the spacing between the two dipole elements and the losses of its external coupling network.

**Figure 1:**
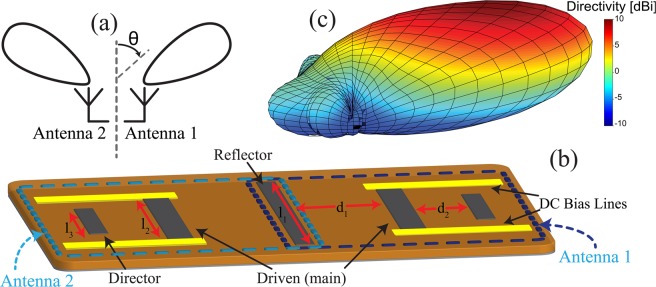
(**a**) Two identical directive infrared antennas placed back to back, can be used to construct an angle sensing detector. (**b**) An example of an angle sensing detector using two Yagi-Uda antenna arrays. Each antenna consists of three dipole elements. The left element in Antenna 1 is the reflector, which is slightly longer than the driven (main) element. The driven element is the middle element connected to the bias lines for measurement purposes. The right most element in Antenna 1 is the director with a length that is slightly shorter than that of driven element. The dimensions shown in this figure are the design parameters that need to be optimized to achieve the desired radiation patterns at the desired operating frequency. (**c**) The three dimensional radiation pattern of a single Yagi-Uda array (Antenna 1 in part (**b**)). Directivity is defined following the standard IEEE definitions^18^. Higher directivity values correspond to regions of higher angular sensitivity in the receiving mode.

In this paper, we present a different approach for designing angle-sensing LWIR detectors with sub-wavelength dimensions. The proposed approach is based on using directional optical antennas such as Yagi-Uda arrays. A simple angle-sensing detector of this type consists of a pair of such directional optical antennas each designed to provide maximum radiation/absorption in a specific direction. This concept is schematically shown in Fig. 1(a). In this case, each of the two antennas (Antenna 1 and Antenna 2 in Fig. 1(a)) is a Yagi-Uda array with a directional radiation/absorption pattern whose peak is directed towards a specific direction in space. In a conventional Yagi-Uda array placed in a homogeneous medium, the direction of maximum radiation is along the direction of the array’s axis. However, placing the antenna at the interface of air and a dielectric as well as adding a ground plane at the bottom of dielectric results in tilting the direction of maximum radiation towards higher elevation angles^24,25^. In the proposed structure, each antenna is coupled to an infrared detector (e.g., a bolometer or an MIM diode). By comparing the relative power absorbed in each detector, one can detect the angle-of-incidence of the IR wave.

 Figure 1(b) shows the topology of the angle sensing LWIR detector. The structure is composed of a pair of Yagi-Uda arrays placed back to back in a symmetric fashion. Each Yagi-Uda array has three dipole elements and to save space, the two arrays share one of their elements (the reflector). This way, the directions of maximum sensitivity (i.e., maximum power absorption) of the two antennas are mirrored with respect to the symmetric plane of the detector. Each Yagi-Uda array uses a bolometer-type IR detector whose resistance variation is proportional to the IR radiation absorbed in the corresponding antenna. This resistance change can be measured by applying a constant bias voltage through the DC bias lines shown in Fig. 1(b), and monitoring the changes in the DC current passing through the bolometer. The responses of the two antennas and the shapes of their radiation patterns can be optimized such that the ratio of the absorbed IR power becomes a one-to-one function of the incidence angle within a wide dynamic range. In our design, the Yagi-Uda arrays are designed to operate at 10.6 *μ*m (28.3 THz) and have radiation patterns whose peaks are directed towards *θ* = ±45° (one of these patterns is shown in Fig. 1(c)). With this design, the ratio of the absorbed power in each antenna (i.e., the detector’s response) is a one-to-one function of the incidence angle over a field of view of ±60° from normal incidence and a dynamic range of approximately 22 dB is achieved.

Most antenna-coupled infrared detectors that use bolometers as the detection mechanism employ different metals to form the body of the antenna and the bolometer. For example, gold can be used to implement the body of the antenna and materials with higher temperature coefficients of resistance (TCRs) such as nickel^26^ or vanadium-oxide^27^ have been used to implement the bolometer. In this work, however, we use a distributed bolometer approach where the body of the antenna also acts as the bolometer. This approach simplifies the fabrication process and reduces the fabrication errors that can be caused by misalignment and nonuniformity of the materials in the device. However, this approach is expected to slightly reduce the response time of the device since the overall size of the bolometer increases. The impact of this effect can be estimated by calculating the response times of detectors designed using these two different approaches. In bolometers, the response time is estimated using their heat capacity divided by their thermal resistivity^2^. Using the thermal properties of gold and nickel as well as their dimensions, we can show that the response time of the proposed distributed-type bolometer detector is approximately three times slower than the same type of detector if it was made primarily out of gold. In this comparison, we assume that the gold detector uses a smaller nickel bolometer, which is placed at the center of the driven element and whose length is one-fifth of the total length of the antenna (0.5 *μ*m). In the current design, the director and the reflector are also made out of the same material as the driven element primarily for the simplicity of the fabrication. However, since they are only parasitically coupled to the main element (i.e., no DC connection between them exists), only the main elements act as the bolometer detectors. The bias lines are made out of gold, which has a lower TCR and lower electrical and thermal resistance. Using Au in bias lines reduces the temperature variations in the bias line and lowers the resistance Johnson noise. The proposed concept for designing angle-sensing IR detector has several advantages over the previously-reported designs (e.g., that reported in ref. ^17^). First, this concept allows for achieving a considerably better dynamic range than previously reported designs. Secondly, using one of the elements of the array as the detector itself simplifies the fabrication process (particularly alignment issues involved in the electron-beam lithography). Additionally, the proposed detector provides a significantly wider bandwidth and better polarization purity compared to other IR-antenna-based angle sensing detectors reported in the literature. Finally, the concept proposed in this paper can be easily expanded to arrays with larger dimensions (e.g., to achieve wider dynamic range and improved sensitivity) or those that use different polarizations (e.g., right- or left-hand circularly polarized).

## Results

### Design procedure

The distributed bolometer antennas used in this design are Yagi-Uda arrays. Yagi-Uda arrays are directional antenna arrays consisting of a few antenna elements parasitically coupled to each other (e.g., see Antenna 1 in Fig. 1(b)). In such arrays, only one of the array elements is driven (or connected to a load) and the other elements are reactively terminated. Often, the driven element is surrounded by a single reflector and one or more director elements. In a dipole-type Yagi-Uda array, the driven element is approximately half a wavelength long and the director and reflectors are slightly longer and shorter than the driven element, respectively^28^. Because of the differences in lengths, the mutual couplings between the driven element and the director and reflector elements will be slightly different. This different coupling causes the currents induced by the driven element on the reflector and directors to have a phase delay that progressively decreases from the reflector to the main element and the directors. The directivity of a Yagi-Uda array can be increased by increasing the number of directors, which in turn increases the overall size of the antenna. In this work, we use an identical pair of three-element Yagi-Uda arrays as the building blocks of the proposed detector shown in Fig. 1(a).

We designed the Yagi-Uda arrays to be fabricated on a silicon dioxide substrate backed with an aluminum ground plane. This ground plane has a thickness of 200 nm, which is approximately 14 times the skin depth of Al at 28.3 THz (*δ* = 15.4 nm). In addition, the 900 nm thickness of the silicon dioxide layer was chosen to maximize the power absorption in the antenna. This value was found by performing full-wave electromagnetic simulations for various substrate thicknesses and monitoring the absorbed power in the bolometer. The entire structure is supported by a silicon substrate. Using this configuration, the length of the driven element of the Yagi-Uda array was calculated to be approximately half a wavelength:

1 $${L}_{{\rm{main}}}=\frac{\lambda }{2}=\frac{{\lambda }_{0}}{2\times \sqrt{{\varepsilon }_{{\rm{eff}}}}},{\varepsilon }_{{\rm{eff}}}\approx \frac{{\varepsilon }_{r}+1}{2}.$$

Here *λ*_0_ is the free-space wavelength (10.6 *μ*m), and *ε*_*r*_ = 4.7 − *j*0.23 is the relative permittivity of silicon dioxide at this wavelength^29^. Using these values, the length of the driven element was determined to be around 3 *μ*m. However, since the antenna material, nickel, is not a perfect electric conductor and silicon dioxide is a very dispersive material around the operating frequency 28.3 THz, the final length of the driven element was determined using this length as an initial value in a fine-tuning process that was conducted in full-wave electromagnetic simulations (see the Methods section). Furthermore, the widths of the dipoles also affect the resonant frequency of the elements used in the Yagi-Uda array. Finally, since the driven element acts both as part of the antenna and the bolometer detector, its width and film thickness should be chosen to ensure that it can tolerate sufficient levels of DC bias current (few tenths of *μ*A) during the measurement without sustaining any damage. Following these considerations, we determined that the width and thickness of 400 nm and 70 nm respectively satisfy these requirements (refer to the Methods section for details). Although using lower width and thickness of the bolometer increases its resistivity and results in higher sensitivity of the bolometer, the fabrication challenges and the fragility of the bolometers with smaller dimensions than these (when subjected to the DC bias current) prevented us from using thinner or narrower bolometers.

**Figure 2:**
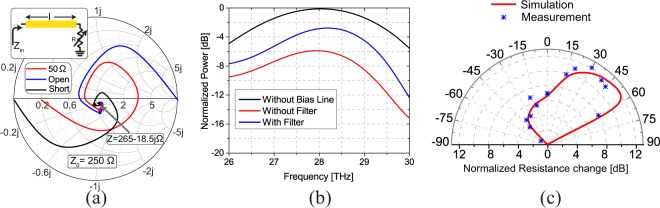
The impact of the bias line on the performance of the Yagi-Uda arrays used in the proposed angle-sensing detector and the radiation patterns of one of the Yagi-Uda arrays in the two-element angle-sensing detector. (**a**) The input impedance of the DC bias line (acting as an IR transmission line) as a function of its length. Three examples are shown where the transmission line is terminated with 50 Ω, open, and short. The input impedance converges to the constant impedance of 265–18.5j Ω as the length is increased. This is the characteristic impedance of the DC bias line at the operating frequency of 28.3 THz (*λ* = 10.6 *μ*m). The dots on each curve correspond to the length of 30 *μ*m. (**b**) The absorbed power in the bolometer (driven element) of a single Yagi-Uda array when the antenna is illuminated with an IR wave arriving from *θ* = 45° for three different situations: (1) The antenna in isolation (no bias line). (2) The DC bias lines are directly connected to the antenna. (3) The DC bias lines are connected to the antenna with an in-line, low-pass IR filter. In all of these simulations, the length of the driven element was optimized to maximize the received power at the frequency of 28.3 THz, when the incident wave comes from the angle of *θ* = 45°. (**c**) The radiation patterns of one of the Yagi-Uda antenna arrays in the optimized IR detector. The radiation is maximized around *θ* = 45°, and its intensity reduces by more than 10 dB for negative incidence angles where the reflector reflects the radiated field.

The other important design parameters are the dimensions of reflector and directors as well as their separations from the driven element. The reflector element behind the driven element should be longer than half of the wavelength to act as an inductively detuned element. When the antenna is analyzed in the transmitting mode (i.e., the main element is driven with a source), the current induced on the reflector leads that of the main element. On the other hand, directors in front of the feed are capacitively detuned elements. The currents induced on these elements lag that of the driven element. This way, the fields radiated by the reflector, the driven element, and the directors interfere constructively towards the front of the array (i.e., a direction from the reflector to the directors). Therefore, such an array generates directional three-dimensional radiation patterns of the type shown in Fig. 1(c). In order to receive a maximal coupling effect from the coupled elements (reflector and directors), they should be placed apart from each other by a distance comparable to the wavelength^30^. To achieve maximum directivity, the reflector should be placed approximately 0.25*λ* behind the main element, and the director should be placed approximately 0.3*λ* in front of the driven element^31^, where *λ* is the effective wavelength calculated using Eq. (1). These dimensions, however, are approximate and constitute values that can be used as initial values in an optimization process that must be conducted using full-wave electromagnetic simulations to achieve the desired response. Following this process, we determined the finalized physical dimensions of a single Yagi-Uda array and provide them in Table 1. The black curve in Fig. 2(b) shows the simulated absorbed power in the bolometer of this antenna as a function of frequency. Observe that with the dimensions reported in Table 1, the Yagi-Uda array achieves maximum power absorption at 28.3 THz (i.e., *λ* = 10.6 *μ*m) when illuminated with an IR wave from *θ* ≈ 45°.

**Table 1:** Final optimized dimensions of the Yagi-Uda antenna arrays used in the proposed angle-sensing detectors. The variables are defined in Fig. 1(b).

*l*_1_	3.75 *μ*m	*d*_1_	1.75 *μ*m
*l*_2_	2.4 *μ*m	*d*_2_	0.8 *μ*m
*l*_3_	1.5 *μ*m	—	—

Although this single Yagi-Uda array has a good directionality, we cannot sense the angle of incidence by using the signal from a single antenna. When using a single antenna, the change in the received power can be due to the change of either irradiance, polarization, or angle of incidence of the incoming wave. In order to design an angle-sensing detector whose response is independent of the level of the emitted irradiance, we placed another identical Yagi-Uda array mirrored along the reflector axis, which is shared between the two antennas. Therefore, one of the antennas is sensitive to the radiation coming from positive angles, while the other one has the maximum sensitivity when the IR wave arrives from negative incidence angles. The distance between the main elements of the two arrays is far enough to have a very limited mutual coupling and a very slight change in the radiation pattern of a single antenna array. The angle sensing detection is possible by measuring the absorbed power in bolometers of both antennas and finding their ratio, which is independent of the irradiance of the incident wave. These absorbed power levels can be measured by applying a constant DC bias voltage across each antenna. To this end, bias lines are connected to the driven elements of Yagi-Uda arrays. In addition to serving the important role of providing a DC bias to the antennas, the bias lines can also impact the IR performance of the antennas. Specifically, each bias line acts as an infrared transmission line the two ends of which are respectively connected to a bias pad and the driven element. From the driven element’s point of view, each of its two ends is connected to infrared transmission lines terminated with an unknown IR impedance at the bias pads. This unknown load that the transmission line is terminated with (*Z* in the inset of Fig. 2(a)) will change the impedance seen at its input (the point where a bias line is connected to one end of the driven Yagi-Uda element, marked as *Z*_*in*_ in the inset of Fig. 2(a)). The loading effect of these two transmission lines impacts the behavior of the driven element and changes its response from that of the isolated Yagi-Uda array discussed earlier. Indeed, the loading effect of the bias lines can cause the effective length of the driven element to be different from what was calculated using Eq. (1). Therefore, the impact of the bias line on the performance of each Yagi-Uda array must carefully be taken into account in the design stage. Furthermore, since each angle-sensing detector uses a pair of Yagi-Uda arrays, having symmetric similar loading effect from the bias lines connected to each array is important. Fortunately, the bias lines are lossy transmission lines at infrared wavelengths. This loss is due to the dielectric loss of silicon dioxide substrate, and the conductor loss of gold at these frequencies. Therefore, if the lengths of the transmission lines are sufficiently long (longer than a few wavelengths), their input impedances will not be a function of the termination impedances at the bias pads. This effect is shown in Fig. 2(a) where the impedances seen at the input of a lossy infrared transmission line are shown on the Smith Chart for different termination loads as a function of the length of the transmission line. The infrared transmission line used to generate these results is the same as one of the bias lines connected to the driven element of the Yagi-Uda arrays shown in Fig. 1(b). The simulated bias line has a film thickness of 100 nm, width of 400 nm, and its length is varied between 0 and 150 *μ*m (*ℓ* in the inset of Fig. 2(a)). This transmission line is placed on the same substrate structure as that of the Yagi-Uda arrays. Observe that for all three termination impedances (short, open, and 50 Ω) the input impedance converges to a value of 265–15.5j Ω for bias lines longer than 30 *μ*m (the dots shown on each line of Fig. 2(a) represents the input impedance for 30 *μ*m). This value is the characteristic impedance of the line and is independent of the load with which the line is terminated. Based on these simulation results, we used DC bias lines with lengths of 50 *μ*m.

Although the designed bias lines provide a constant impedance seen by the driven elements of the antennas, this impedance is different from the open load condition, in the absence of the bias lines. To examine this loading effect, the absorbed power in a single Yagi-Uda antenna is simulated for two cases, with and without the bias lines, using full-wave EM simulations. As shown in Fig. 2(b), the absorbed power of the antenna is reduced by 6 dB (a factor of 4) when the driven element is connected to the bias lines. This reduces the detectivity of the detector by a factor of 4. This loading effect can be reduced using a simple low pass filter. Here, we designed a simple one element low pass filter using a quarter wavelength transmission line placed a quarter wavelength away from the antenna. Using this filter in the DC bias line increases the input impedance seen at the input of the bias line from 265–15.5j Ω to 570–42j Ω. This higher input impedance helps reduce the loading impact of the bias lines on the Yagi-Uda array. Figure 2(b) also shows the absorbed power in a single Yagi-Uda array when it’s biased with DC bias lines having this low-pass filter. Although adding the filter does not completely remove the loading effect of the bias lines, it increases the absorbed power by 3 dB (a factor of two improvement in the power absorbed by the antenna when directly attached to the bias line).

The radiation patterns of one of the Yagi-Uda arrays of the finalized detector, in the presence of filters and bias lines, is shown in Fig. 2(c). The maximum direction of peak radiation/absorption is towards *θ* = 50° and very low absorption is expected for negative angles of incidence. The deviation of the direction of maximum simulated directivity from 45° in the initial design of the detector to 50° in the final design is due to the effect of the bias lines. This small difference does not impact the final angle-sensing detection of the detector presented in the device characterization section. Figure 2(c) also shows the measured radiation patterns of a single antenna in the detector. The discrepancies observed between the measured and simulated radiation patterns are attributed to several factors in the simulation, fabrication, and measurement. First, the material parameters used in the simulations were were taken from experimental characterizations performed by others and reported in the literature^29,32–34^. We expect that the constitutive parameters (i.e., complex refraction index) of the materials used in our fabricated prototype may be slightly different from those assumed in the simulations due to the different fabrication procedures used. Also, the simulations were performed assuming that the supporting silicon dioxide layer and the ground plane underneath it are infinite in extent. This choice was made to simplify the simulation process of the detector. In reality, however, the fabricated prototype has finite dielectric and ground plane dimensions. In addition, due to the finite precision of the fabrication processes used, the precise dimensions of the fabricated prototype are slightly different from those used in the simulations. Moreover, the readout circuitry and the incident wave used in the experiment are not ideal as assumed in the simulation process. The laser power variation, noise of the bias voltage source, and the alignment of the laser beam with the center of the antenna are some of the uncertainties that may have contributed to the differences between the simulation and measurement results reported in Fig. 2. However, these differences do not significantly impact the angle sensing behavior of this detector as we show later in this paper.

**Figure 3:**
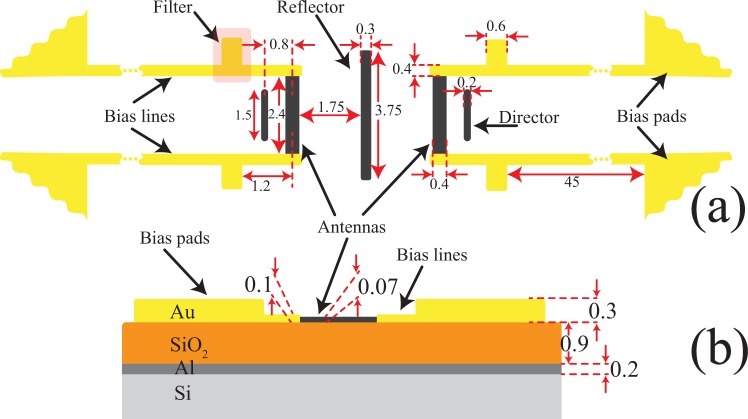
Topology of the final design of the angle-sensing detector. (**a**) The device is composed of two, back-to-back Yagi-Uda antenna arrays that share the same reflector element. The antenna elements are all made out of nickel and the center element in each array also acts as the bolometer. The bias lines are made of gold, which has a lower TCR compared to nickel. (**b**) The cross-sectional view of the detector shows all the layers and their thicknesses in the substrate and antenna. Au is the material used to fabricate the bias lines and pads. However, due to wirebonding procedure, pads are made of a thicker Au film. All dimensions are in *μ*m.

The topology of the finalized design with all dimensions marked is shown in Fig. 3(a), and the cross-section view of the detector is shown in Fig. 3(b). As shown in these figures, bias lines are connected to bias pads for measurement purposes. The fabricated bias pads are made of thicker deposited gold layers (300 *μ*m) than the bias lines (100 *μ*m), since this is the minimum film thickness that allows for having a reliable wirebonding during the characterization.

### Device characterization

**Figure 4:**
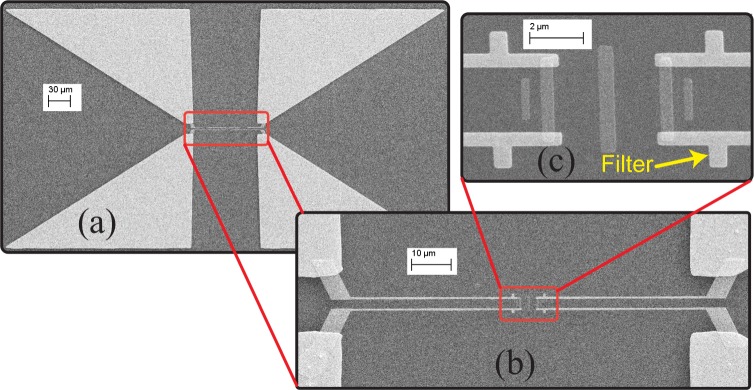
SEM image of the fabricated device prototype. (**a**) The detector and bias lines are at the center of this image, and connected to four large bias pads, which were wirebonded and connected to DC bias source during the measurement. (**b**) Bias pads are connected to bias lines. The length of each bias lines with its inline filter is 50 *μ*m. (**c**) The magnified image of the two Yagi-Uda arrays and part of the bias lines with short stub filters is shown in the inset. The whole antenna part is fabricated in a single lithography step, while the bias lines are formed using a separate step.

The angle-sensing detector discussed in the previous section was fabricated using standard nano-micro-fabrication techniques, with three steps of e-beam lithography (see Methods section for a detailed description). The scanning electron microscope (SEM) images of the fabricated device are shown in Fig. 4. Figure 4(a) shows four large triangular bias pads, which were wirebonded to a sample holder, and connected to a constant bias voltage. These bias pads are directly connected to the bias lines, as shown in Fig. 4(b). The magnified image of the detector in Fig. 4(c) shows the antennas’ elements and part of the bias line with the IR filter. Since a distributed bolometer structure is used, and also parasitic elements are fabricated in the same step as the driven element, no misalignment exists between the two Yagi-Uda arrays, and their elements.

**Figure 5:**
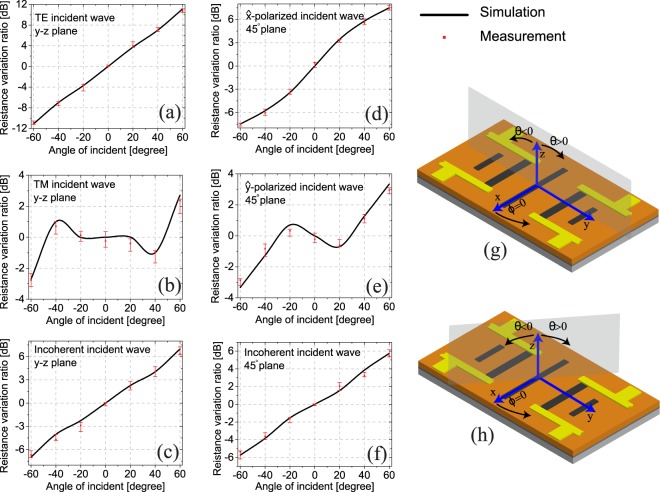
Simulation and measurement results of the angle sensing detector for various polarizations and planes of incidence. (**a**–**c**) The response of the detector for (**a**) $\widehat{x}$-polarized, (**b**) $\widehat{y}$-polarized, and (**c**) incoherent polarizations when the wave is incident within the *y* − *z* plane. Here, the angle of incidence was measured from the *z* axis. + and − *θ* values are defined in part (**g**). (**d**–**f**) The results for (**d**) $\widehat{x}$-polarized, (**e**) $\widehat{y}$-polarized, and (**f**) incoherent polarizations when the wave is incident from a plane that makes a 45° angle with the *x* axis. + and − *θ* values are defined as shown in part (**h**). Error bars in these figures are the results of the measurements of few different devices, and also multiple measurements of a single device with different power of the laser.

The response of the detector was simulated for various angles of incidence in different axes of rotation, and different polarizations. Specifically, the transverse electric (TE), transverse magnetic (TM), and incoherent polarization illuminations were considered. The details of numerical electromagnetic simulations employed are discussed in the Methods section. The response of the fabricated detector was measured by illuminating it with a *CO*_2_ laser at 10.6 *μ*m. Fig. 5(a–f) show the simulated and measured responses of the detector for various polarizations of incidence and planes of rotations (Fig. 5(g,h)). The measured response for each angle of incidence and various polarizations were repeated for a few devices and various radiated powers of the laser. The variations of these measurements for a single data point are shown by error bars. The error bars show the maximum and minimum of the measured data points. These variations in the response of the device are the result of the non-idealities of the fabricated devices and the measurement process. In other words, the response of various devices could vary due to the imperfection of each fabrication step, and the presence of noises during each measurement, and the fluctuation of laser’s power.

As shown in Fig. 5(a), the device has symmetric response with a wide dynamic range of 22 dB for TE polarized waves incident within the *y* − *z* plane. In this incidence plane, the TE incidence has an electric field component oriented along the $\widehat{x}$ direction. For this plane of incidence, the polarization of the antenna and that of the TE incident wave are matched and the detector has its highest sensitivity. In this case, an excellent agreement between the measured and simulated responses is observed. Since the Yagi-Uda antennas used in the construction of this detector consist of linearly-polarized dipole antennas, the response of the detector is expected to be polarization sensitive. Specifically, under ideal operational conditions, the detector is not expected to respond to incident waves whose polarizations are orthogonal to those of the antennas used in the detector. Therefore, for the TM polarization in the *y* − *z* plane of incidence, the receiving efficiency of the Yagi-Uda array is significantly reduced. However, for TM polarized waves incident within the *y* − *z* plane, the power absorbed in the bias lines increases since they are parallel with the polarization of the incident wave. Moreover, for TM polarization of incidence in the *y* − *z* plane, the electric field has both a $\widehat{y}$ and $\hat{z}$ component. Thus a slot mode may be exited between the different dipoles constituting the Yagi-Uda array and some level of EM energy can still be absorbed by the antenna. However, full-wave EM simulations suggest that the energy absorbed in the detectors is significantly enhanced (up to a factor of 10×) when the bias lines are present in the detector. As a result of these different factors, the detector response is no longer a monotonic function of the incidence angle, *θ*, as shown in Fig. 5(b) and the dynamic range is smaller. In addition, for incoherent polarization, the angle sensing response is almost the average of the responses of the device for the TE and TM modes of incidence, as seen in Fig. 5(c). Figure 5(d–f) show the responses of the device when the plane of incidence of the incoming wave makes a 45° angle with the *x*-axis (shown in Fig. 5(h)). In this incidence plane, the response of the device was measured for both the co-polarized (polarization of the incident wave along the $\hat{x}$ direction) and the cross-polarized (polarization of the incident wave along the $\widehat{y}$ direction). For both the co-pol and the incoherent polarizations, the device’s response is still a one-to-one function of the incidence angle, albeit with a reduced dynamic range. Observe that the detector has the highest dynamic range and monolithic response for only the incident waves that are polarization matched to the Yagi-Uda antennas used. However, the imperfect response of the detector for other polarizations does not limit the applications of the detector. In a practical application, a polarizer can be placed in front of it to ensure that the detector is illuminated with the correct polarization. Alternatively, the detector can be used without such a polarizer at the expense of having a reduced dynamic range (see Fig. 5(c)). Also, different arrangements of angle-sensing detectors of this type as well as other angle-, polarization-, and frequency-sensing detectors may be used collectively in new types of sensing/imaging devices that can be used to sense other attributes of incoming waves beyond its irradiance. In such systems, new sensing/imaging algorithms have to be developed to take advantage of these unique individual sensor responses and perform sensing and imaging in visually degraded environments.

In addition to the angle sensing response of the detector, other figures of merit (FOM) were also measured to help compare the response of a single antenna used in this detector with those of other published antenna-coupled infrared detectors. In these measurements, signal to noise ratio (SNR) was determined to be 420 when the device was illuminated from the direction of maximum sensitivity (i.e., *θ* = 50° as seen from Fig. 2(c)). The noise equivalent power (NEP) was measured to be 0.36 nW, and the normalized detectivity (D^*^) was calculated to be $$4.25\times 1{0}^{5}\,\,{\rm{cm}}.$$$$\sqrt{{\rm{Hz}}}.{{\rm{W}}}^{-1}$$.

## Discussion

A detector consisting of two directional infrared antennas was used to sense the angle of incidence of the incoming IR radiation, *θ*, using amplitude-only, non-coherent measurements. The directional antennas used to design this detector are planar Yagi-Uda arrays consisting of three infrared dipole antennas (one driven element, a reflector, and a director). Each Yagi-Uda IR array is sensitive to both the intensity and the angle of incidence of the incoming IR radiation. By measuring the ratio of absorbed power in the two mirrored antennas, one can detect the angle of incidence of the incoming radiation independent of its intensity, as long as the incident power density is high enough to allow for the detection of the signal. Therefore, our proposed device can sense both the angle of incidence and the irradiance of the incoming wave simultaneously. The use of only two compact Yagi-Uda arrays in this device allows for keeping the occupied area of the device smaller than a quarter of wavelength squared. To reduce the fabrication complexity, we used a distributed bolometer topology where the driven element of each Yagi-Uda array also acts as a microbolometer. This was implemented by using nickel to fabricate the antenna elements and fabricating the bias lines with gold, which has a lower TCR value. To reduce the impact of the DC bias lines on the performance of the IR detector, long bias lines with an embedded, first-order, low-pass IR filter were employed. This arrangement provides a constant loading effect on the driven element regardless of the shapes and the sizes of the bias pads and the loads connected to them. The response of the detector shows an excellent agreement with the simulated response and a dynamic range of 22 dB was achieved from a detector that occupies an area of 6 *μ*m × 3.75 *μ*m.

This newly proposed angle sensing detector has a number of important advantages over other IR-antenna-based angle-sensing detectors reported in the literature, including a work reported previously by our group^21^. First, due to the use of directional antennas in this detector, the dynamic range has been increased significantly (from 10 dB in ref. ^17^ to 22 dB). Moreover, the same design concept presented in this work can be easily extended to more directive antennas, such as larger Yagi-Uda antenna arrays to further increase the dynamic range and the capture area of the detector. Secondly, the proposed detector is less sensitive to cross-polarized radiation and operates over a wider bandwidth compared to the device reported in ref. ^17^. Finally, the design and fabrication process of this detector is simplified compared to that reported in ref. ^17^.

In the reported prototype, we used nickel as the bolometer and silicon dioxide as the substrate for the simplicity of the fabrication of the antenna and perform proof-of-concept experimental demonstrations. The performance of this device can be enhanced by employing more complicated fabrication techniques. For example, one can use higher TCR bolometers^35,36^, lower loss substrates^37,38^, or thermally isolate the microbolometers from the substrate^39,40^. Additionally, rather than using a bolometer, other detectors such as MIM diodes^8,9^ can be used. These techniques can be used to improve the response time and detectivity of the device. Since our goal in this work was to present a proof-of-concept experimental demonstration of the underlying angle-sensing concept, we did not pursue these more complicated fabrication techniques. Nevertheless, the measured performance of the device (e.g., its detectivity) is comparable to the performances of other antenna-coupled infrared detectors that use bolometers^26,41,42^.

## Methods

In this section, details of simulation, fabrication, and measurement methods are presented.

### Computer simulations

The device response was simulated and the device was optimized using CST Microwave Studio, a commercial full-wave electromagnetic simulator. The first step in these simulations was to import the optical properties of different materials used in the structure of the detector into the software. These frequency-dependent properties were obtained from the literature^29,32–34^. Then, the device structure as shown in Fig. 3(a,b) was implemented. In these simulations, the boundary conditions on the bottom, top, and side surfaces were respectively perfect electric conductor (PEC), open add space (open, perfectly matched layer), and open boundaries. A plane wave with a given polarization and angle of incidence was defined as the excitation source of this problem.

The time-domain solver of CST Studio was used to calculate the input impedance of the DC bias lines (see Fig. 2(a)) and to calculate the radiation pattern of the antenna shown in Fig. 2(c). Time-domain simulations in CST Studio were also used to calculate the total infrared power absorbed in different parts of the antenna (e.g., the bolometers, bias lines, etc.) for a given incident plane wave. This calculated IR absorbed power was then used as a source in the thermal solver of CST Studio to calculate the temperature change in different parts of the antenna. Knowing the TCR of all materials, and the average temperature of each part, the resistance variations for each side of the detector were calculated. The output ratios shown in Fig. 5 are the ratios of the resistance changes. These simulations were repeated for various directions and polarizations of the illuminating plane wave.

### Fabrication

The device was fabricated using standard clean room micro and nano-fabrication techniques in Wisconsin Center for Applied Microelectronics (WCAM). The fabrication was started with a three-inch high resistivity silicon wafer, which was cut into small pieces after depositing aluminum, and silicon dioxide. The 200 nm aluminum, which serves as the ground plane was deposited using DC sputtering at the continuous mode of rotation to provide better uniformity. The 900 nm-thick *SiO*_2_ dielectric was deposited using plasma enhanced chemical vapor deposition, at a temperature of 350 °*C*, and with a deposition rate of 400 A°/min. Then, the wafer was cut into 9 mm × 9 mm pieces for the rest of the fabrication.

Since the minimum feature size of the designed detector is 200 nm (width of the directors), the device was patterned using electron beam (e-beam) lithography. Three steps of e-beam lithography were used to pattern the antenna, bias lines, and bias pads, respectively. In each step of lithography, a bilayer e-beam resist consisting of polymethyl methacrylate (PMMA) and its copolymer (MMA) was used to provide a great undercut for more reliable lift-off process. After patterning the desired structure, samples were developed with methyl isobutyl ketone (MIBK) in combination with Isopropyl alcohol (IPA) (1:3), followed by descum process. Then, the desired metal was deposited with an electron beam evaporator. In the first step of lithography, 70 nm-thick nickel was deposited to patterns the bolometers. The 10/100 nm and 10/300 nm titanium/gold were deposited respectively in the second and third steps for bias lines and pads. The deposited 10 nm titanium in these steps helps the adhesion of gold to silicon dioxide substrate. After metal deposition, the samples were placed inside a tank of acetone with applying ultrasonic vibration to remove extra metals and e-beam resist (lift-off process). When all the steps of the fabrication were completed, each device was wire bonded to a sample holder using a gold ball wire bonder, which provides connections to measurement setup. Different steps of the fabrication are included in Fig. S1. of the supplementary materials.

In order to detect the resistance change of the bolometers, a DC bias current (in the order of a few tenths of mA) was applied to the device. We experimentally observed that Ni bolometers with thicknesses of lower than 70 nm and widths of lower than 400 nm cannot tolerate this amount of current and would burn as soon as we applied the bias voltages. The exact thickness and width of the bolometers were experimentally determined by fabricating several prototypes of angle sensing detectors with different thicknesses and widths of Ni bolometers. Using this process, we selected the minimum thickness and width that could reliably handle the applied bias voltage and current.

### Measurement

The response of the detector was characterized under illumination by a *CO*_2_ laser (L4S from Access Laser) at a wavelength of 10.6 *μ*m, for various angles and polarizations of incidence. The polarization of the incoming wave was dictated by passing the laser through a wire grid polarizer (Thorlab WP25M-IRC), which was mounted on a rotational stage. The angle of incidence was determined by rotating the device. The device was placed on two rotational stages on top of a 3D moving stage. One of the rotational stages controlled the angle of incidence, while the other controlled the plane of rotation for the two states shown in Fig. 5(g,h). The angle sensing response was measured by rotating the device every 20°. The 3D stage was used for aligning the device to the laser beam in different measurements.

After the wire grid polarizer, the laser beam was passed through a power beamsplitter (Thorlabs ZnSe plate), which directs half of the beam to a power meter to measure the power incident on the device in various measurements. Although this power measurement monitored the laser power in various measurements, the angle sensing response of the detector is independent of the incident power level. However, the measured power was used to calculate the irradiance of the incoming wave for NEP measurements. The other half of the beam was passed through an SR540 Stanford Research System’s chopper. The chopper modulated the laser beam by mechanically chopping it at 400 Hz. The chopper and output signal were connected to a 7230 Signal Recovery lock-in amplifier, which demodulated the output signal by detecting the frequency of the chopper. The output signal connected to the lock-in amplifier comes from the output of an SR570 Stanford Research System current preamplifier, which amplifies the current variations passing through the device. The amplifier had embedded high pass filter, which filters out the DC, and the low-frequency components of the current and only passed the high frequency current components, which is equivalent to current variation in the device in the presence of the laser beam. The amplifier was placed in series to a bias voltage to apply a constant DC bias voltage on both sides of each antenna. The schematic of the measurement setup is shown in to the Fig. S2 of the Supplementary Materials.

The SNR was measured by dividing the measured output signal to the measured noise of the detector determined by lock-in amplifier. The measured noise is the combination of the resistor Johnson noise of the device and flicker and shot noise of the bias voltages. This calculated SNR was used to find the value of NEP, which is defined as the minimum laser power that can generate a detectable output signal from the detector. This value can be calculated by dividing the power incident on the detector (*ϕ*_*e*_) to the SNR. *ϕ*_*e*_ was determined by the product of the incident power intensity and the size of the detector. Using the measured power of the laser with IR power meter and knife-edge method^43^ to find the beam spot size on the place of the detector, the power intensity of the laser was calculated and used to find the value of NEP. Then the normalized detectivity, which is defined as ${D}^{* }=\sqrt{{A}_{eff}\times BW}$/*NEP* was estimated. In this formula, *A*_*eff*_ is the detector area, and the *BW* is the electrical bandwidth of the measurement setup, which was 0.1 Hz in our measurements.

## Supplementary information

Supplementary Information.
