# Supplementary material for: Wide Dynamic Range, Angle-Sensing, Long-Wave Infrared Detector Using Nano-Antenna Arrays
Source: Sci Rep. 2020 Feb 12;10:2488. doi: 10.1038/s41598-020-59440-2 (PMC7016179; doi:10.1038/s41598-020-59440-2)
Supplement: Supplementary file 1 — Supplementary Information. [file 41598_2020_59440_MOESM1_ESM.docx]

Wide Dynamic Range, Angle Sensing, Long-Wave Infrared Detector Using Nano-Antenna Arrays

Elham Mohammadi ^1^, Mohammad Ghaffari ^1^ Nader Behdad ^1^

^1^ University of Wisconsin-Madison, Department of Electrical and Computer Engineering, Madison, WI, 53706, USA

^*^ Corresponding author Email: behdad@wisc.edu

**Supplementary Information**

This file contains 2 supplementary figure.

Captions and legends are also included.

**Fabrication Procedure:**

**Supplementary Figure S1. Fabrication Process.** Different steps of the fabrication, including deposition of various materials, and three steps of e-beam lithography followed by lift-off.


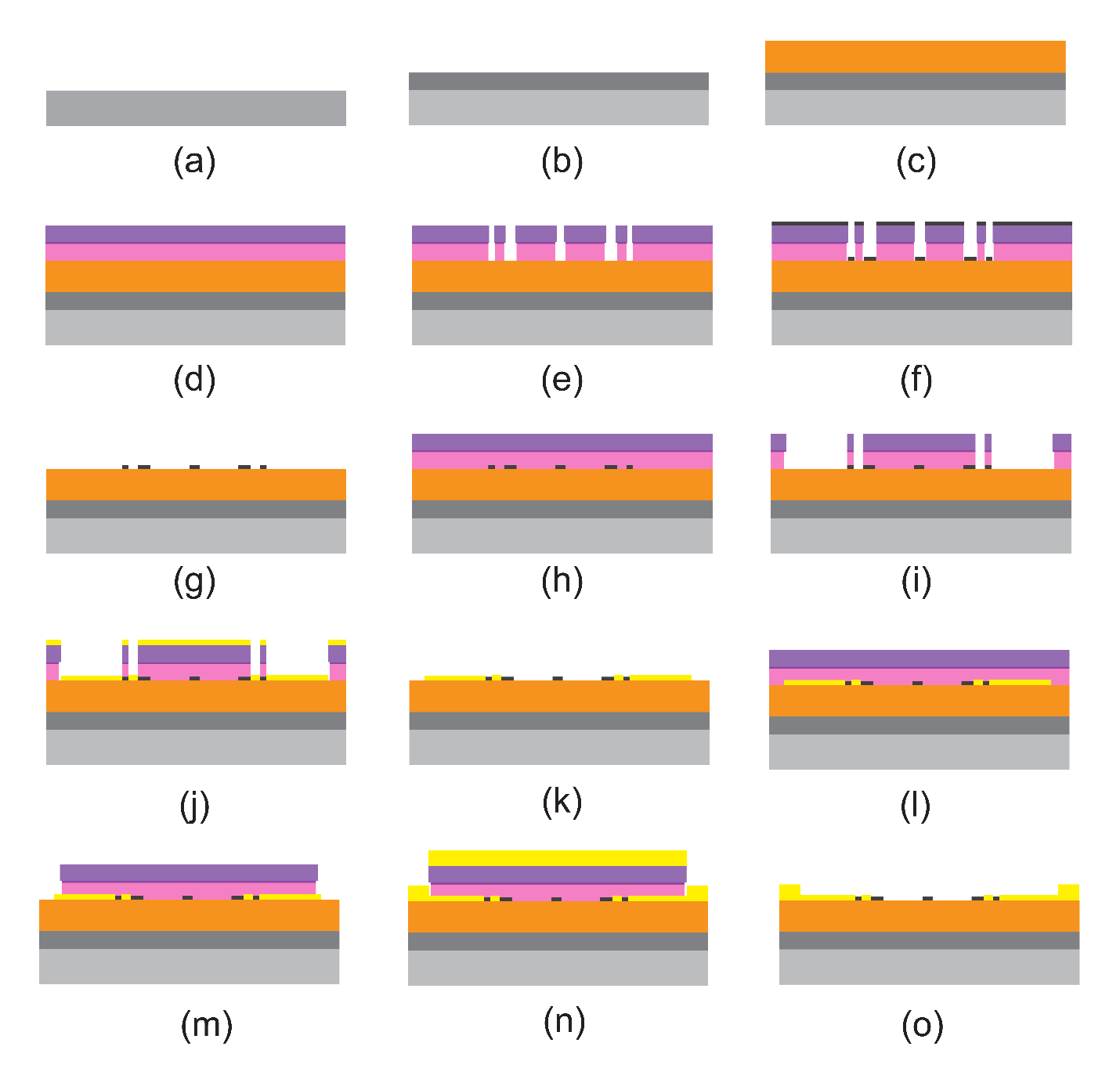


1. Cleaning a 3” wafer, using acetone, Isopropyl alcohol (IPA), Deionized (DI) water.
2. Deposit 200 nm Aluminum, using DC sputtering. Then, cut the wafer into 9 mm by 9 mm.
3. Deposit 900 nm silicon dioxide using plasma enhanced chemical vapor (PECVD) deposition, at temperature of 350^o^ C.
4. Spin coating the E-beam resists (MMA/PMMA): The first step is spin coating the 350 nm-thick MMA, which has higher developing time compare to PMMA. Then, the coated sample was baked for 60 s at temperature of 150^o^ C. The next step is spin coating the sample with 350 nm of PMMA and pre-back it for another 60 s at 180^o^ C. In both steps of spin coating, first the device was spun for 10 s at the speed of 500 RPM and 500 ramp which helps the resist spread over the sample. Then, we span it at the speed 4000 RPM and 1000 ramp for 30 seconds.
5. After coating the sample with e-beam resist, e-beam lithography was done to pattern Yagi-Uda array. The E-beam lithography was done using an SEM tool with the voltage of 30 KV and a NPGS software with the magnification of 1000.

Various doses were used for patterning different parts of the antenna. For example, higher dose was used for patterning the director part, which is narrower (200 nm) compared to the rest of the design. The optimum doses for different part of the device may vary with the calibration of the E-beam tool, and should be found by careful dose measurement steps (i.e. trying multiple doses and finding the ones which result the best device for different parts of the device).

After lithography, the sample was developed using methyl isobutyl ketone (MIBK) in combination with IPA (1:3), for 45 seconds. In this step, all the exposed part during the lithography were removed. Then, the sample was rinsed using DI water. To remove any attached resist to the substrate, the descum process using reactive ion etching was followed by all developing steps.

1. Depositing 70 nm nickel using electron beam evaporation tool with the rate of 0.5^ͦ^A/s.
2. Lift off process: extra resist parts were removed using acetone.
3. The e-beam resists were coated for the second step of lithography, as explained in the step (d).
4. E-beam lithography for patterning the bias lines followed by developing and descum. The magnification of SEM in this step was 900.
5. Deposition of 10 nm titanium and 100 nm of gold, using electron beam evaporation with the deposition rates of 0.2 ^ͦ^A/s for titanium and 1 ^ͦ^A/s for gold. The titanium was added to increase the adhesion of gold to silicon dioxide.
6. Lift off process with acetone.
7. Spin coating the e-beam resist for the third step of lithography.
8. E-beam lithography for patterning the DC bias pads and developing of the exposed resist. Here, we used higher aperture size in SEM tool, which resulted in higher current and increased the speed of writing the pattern.
9. Depositing 10 nm of titanium with 300 nm of gold (the same deposition rates as in the step (j)).
10. Lift off process with acetone.

At the end of these steps of the fabrication, the device was ready for measurement. Therefore, the pads were wire bonded using gold ball wire bonder to a sample holder. In this step, the device was placed on a heater at temperature of 120 ^ͦ^C. The gold wires on the tool had a diameter of 25 μm, and we adjusted the size of gold balls to be around three times of the wire thickness.

**Measurement Setup:**

**Supplementary Figure S2. Measurement setup.** The measurement setup consists of an infrared CO_2_ laser, wire grid polarizer to provide desire polarization, beam splitter, power meter, mechanical chopper, DC bias voltage in series with a current preamplifier, and a lock-in amplifier. In each step of the measurement, the power of the incident laser beam was measured using the power meter.

**
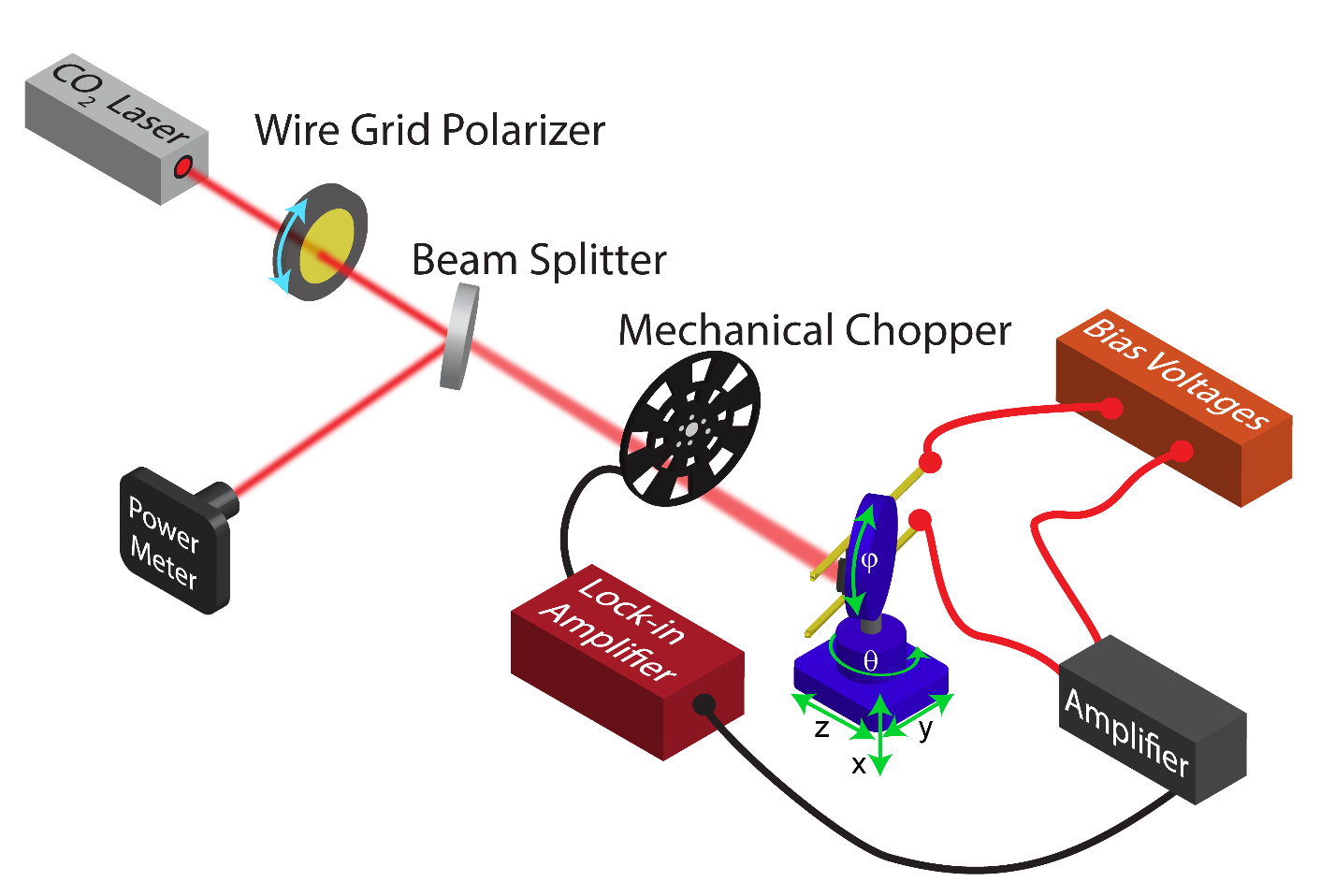
**

Measurement setup includes:

1. CO_2_ laser: The laser provides a randomly polarized infrared beam at wavelength of 10.6 μm.
2. Wire grid polarizer: The polarizer converts the random polarization of the laser to a linear polarization. By rotating the wire grid polarizer, one can get the desired polarization such as TE or TM.
3. Beam splitter: Beam splitter splits the incoming beam into two parts. One part goes to the power meter and the other is incident on the device.
4. Power meter: This device measures the power of the incident beam. This measured value can be used for calculating the detectivity and for measuring the fluctuations of laser power.
5. Mechanical chopper: The incident laser beam was mechanically chopped at a frequency of 400 Hz, which modulates the input signal of the system. The chopper was connected to the lock-in-amplifier, which detects the modulation frequency.
6. Stage: the device was mounted on a 5D stage. The stage can move in *x*, *y*, and *z* dimensions to align the center of the detector to the incidence laser beam. Furthermore, rotational stage of θ determines the angle of incidence and rotational stage of φ rotates the device in its plane to be able to measure the response in various configurations (i.e., ass shown in Fig. 5(h)).
7. Bias Voltage: During the measurement, both sides of each antenna were connected to a constant bias voltage and the current passing through the device was monitored.
8. Amplifier: The current variation in the bolometers were amplified and the lower frequency part of the current such as DC and flicker noise were filtered out.
9. Lock-in-amplifier: The output of the amplifier was connected to the lock-in-amplifier whose job was to demodulate this output by the frequency of the chopper.
